# Rapid extrication of entrapped victims in motor vehicle wreckage using a Norwegian chain method – cross-sectional and feasibility study

**DOI:** 10.1186/1471-227X-14-14

**Published:** 2014-07-03

**Authors:** Sabina Fattah, Anne Siri Johnsen, Jan Einar Andersen, Trond Vigerust, Terje Olsen, Marius Rehn

**Affiliations:** 1 Department of Research and Development, Norwegian Air Ambulance Foundation, P.O box 94, 1441 Drøbak, Norway; 2 Anaesthesia and Critical Care Research Group, Faculty of Health Sciences, University of Tromsø, Tromsø, Norway; 3 Department of Health Studies, Faculty of Social Sciences, University of Stavanger, Stavanger, Norway; 4 Department of Anaesthesiology, Oslo University Hospital Ullevål, Oslo, Norway; 5 Norwegian Air Ambulance, Dombås base, Lørenskog, Norway; 6 Department of Anaesthesia and Intensive Care, Akershus University Hospital, Lørenskog, Norway

**Keywords:** Rapid extrication, Road traffic accident, Road traffic injuries, Rescue services, Emergency medical services, Emergency medicine, Entrapped victims

## Abstract

**Background:**

Road traffic injury (RTI) is a global problem causing some 1,2 million deaths annually and another 20–50 million people sustain non-fatal injuries. Pre-hospital entrapment is a risk factor for complications and delays transport to the hospital. The Rapid Extrication (RE) method combines winching and cutting of both front poles and utilising two larger vehicles to pull car wreckage apart to extricate patients. A previous study indicates that RE is an efficient alternative to previously existing methods.

**Methods:**

All Fire Departments in Norway were questioned on: background, frequency of training, use and implementation of the method, protocol and equipment. Times used for extrication from motor vehicle wreckage were measured at the National Championship in RE. Questionnaires presented to participants asked about frequency of training, inter-disciplinary cooperation and self-perceived safety for both providers and patients on a 1–7 Likert scale (1 - worst and 7 - best).

**Results:**

Participating Fire Departments use RE in 95% of cases on passenger cars and 77% of cases on larger vehicles. Teams participating in the National Championship scored self-perceived security of crew as median 7 and IQR (6, 7), patient safety 7 (6, 7), communication between personnel 7 (6, 7), teamwork 7 (6, 7), and how well the technique functioned 7 (6, 7).

All teams had extricated and transported the patient into the ambulance within 20 minutes.

**Conclusion:**

Interdisciplinary and regular training of RE can lead to safe extrication of a critically injured patient in less than 20 minutes and may be life saving.

## Background

Injury is a major cause of death and disability worldwide. In many instances the prompt provision of emergency care and rapid movement of injured victims from the scene can save lives, reduce the incidence of disability and improve patient outcomes [1]. Road traffic injury (RTI) is a global public health problem causing some 1,2 million deaths yearly and another 20–50 million people sustain non-fatal injuries [2].

When a patient is entrapped in motor vehicle wreckage, extrication needs to be performed both quickly and safely to reduce time to definitive care. Furthermore, pre-hospital entrapment is a significant risk factor for developing complications such as deep vein thrombosis and pulmonary embolism [3].

**Figure 1:**
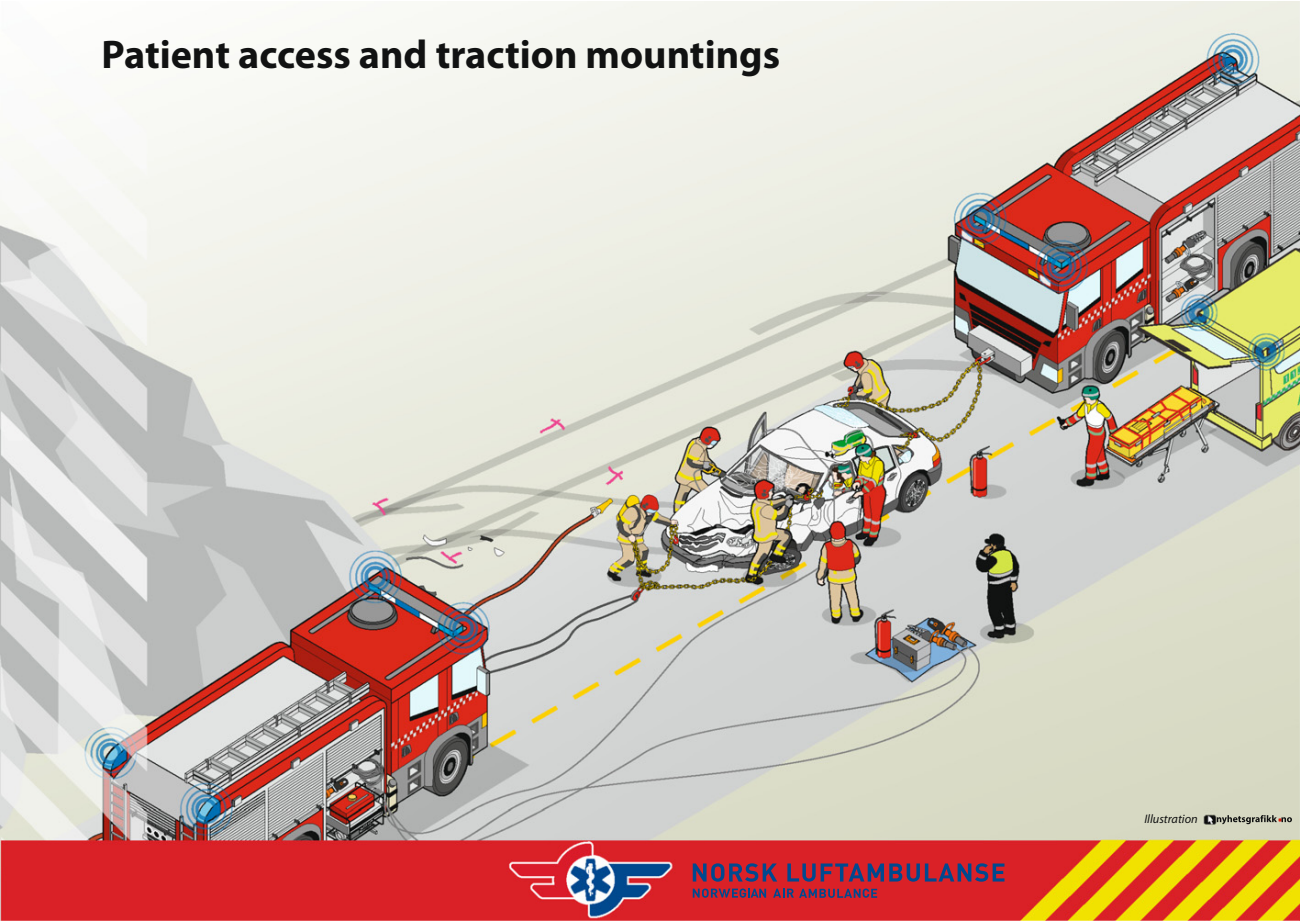
Rapid extrication technique.

**Figure 2:**
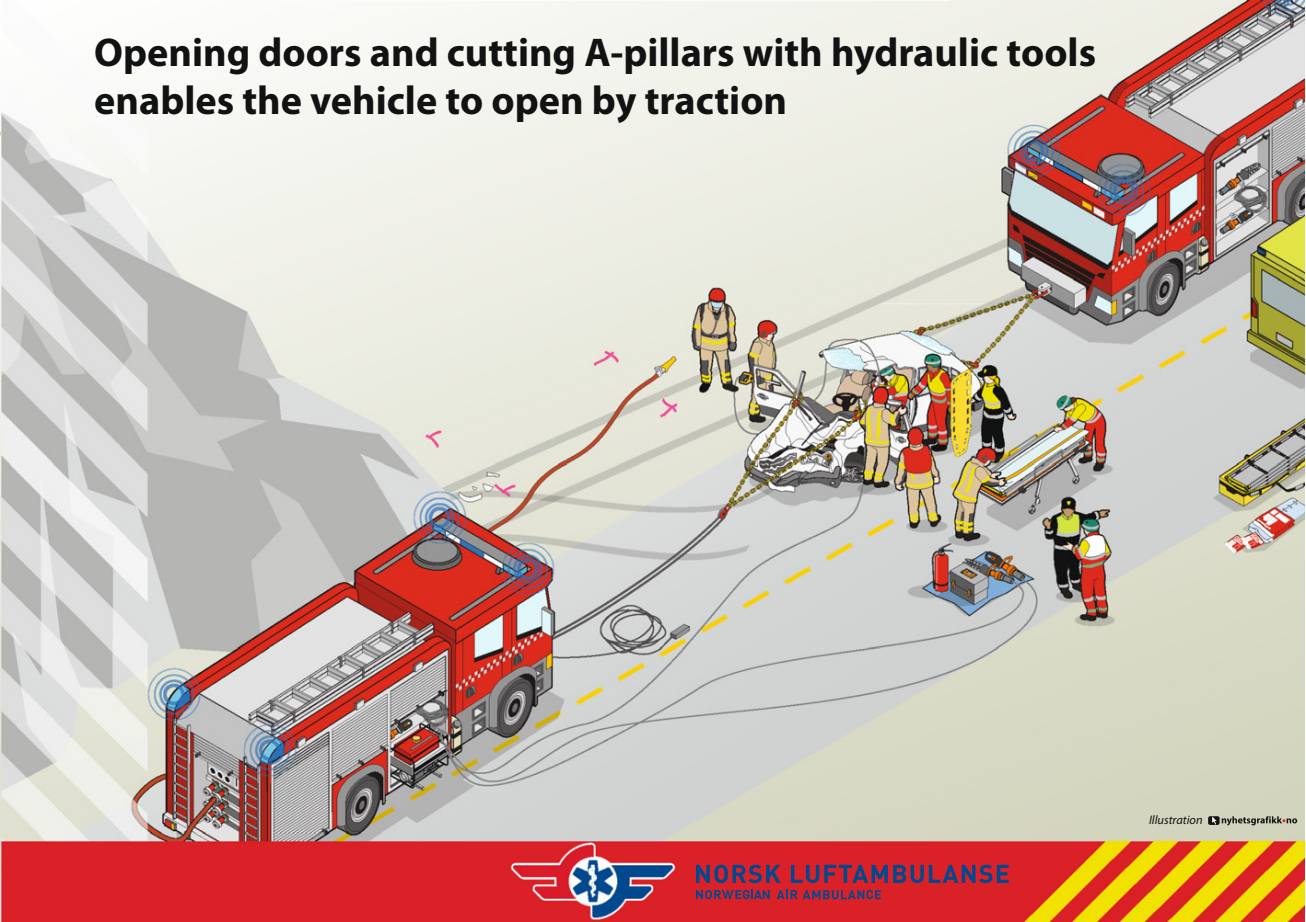
Rapid extrication technique.

Individuals in the Oslo-Akershus Emergency Medical Services (EMS) and Fire Departments developed the Rapid Extrication method in 1998. A previous study indicates that this is a more time efficient alternative to previously existing methods whilst maintaining patient and crew safety [4]. The Norwegian Air Ambulance Foundation (NAAF) has refined the method over the last years and now teaches this as the Rapid Extrication chain method (RE) (c.f. Figures 1 and 2, fact box and Additional file 1). The method is to be used in situations where the patient is entrapped in vehicle wreckage. The tools needed for the method as it is taught by the NAAF are two vehicles; one front and one behind to provide traction and pull. Hydraulic tools and chains are also needed. RE was introduced in the Interdisciplinary Emergency Service Cooperation (TAS) courses in 2002 [5,6]. Some 16500 participants from fire departments, police and EMS in Norway have participated in the more than 450 TAS courses held so far.

**Fact box** – role of different Rescue Services in the use of Rapid Extrication chain method

• **Fire and Rescue:** responsible for safety, hydraulic tools, chain and winching of the car.

• **Police** (Incident Commander): responsible for communication and scene management.

• **Emergency Medical Service:** ambulance/paramedic is responsible for medical examination and triage. One personnel participates in the transportation out of the motor vehicle wreckage.

The main goal is that all rescue personnel coordinate their work, communication, cooperate and understand each others roles on-scene.

The aim of the study was to investigate to which extent the method is implemented in Norway and to investigate how trained teams consider the feasibility of RE.

## Methods

### Cross-sectional survey

The Norwegian Fire Academy and Directorate for Civil Protection and Emergency Planning provided e-mail addresses for all 298 Norwegian Fire Departments. These services were asked to reply to an electronic questionnaire (c.f. Additional file 2). The first request for participation was sent in February 2013 and three reminder e-mails were sent before data collection ended in May 2013. The questionnaire asked for background of the service, frequency of training and use of the technique on both passenger cars and larger vehicles, when the method was implemented and where they had learned the method.

### Feasibility study

The study at the National Championship in RE was performed in September 2012. Participating teams consisted of at least one individual from each of the services: fire, police and EMS. We measured the time expenditure of these teams in the extrication of a simulated patient from motor vehicle wreckage. Questionnaires (c.f. Additional file 3) were presented to the teams after completion of the competition. These contained data on training frequency, how the method worked during the exercise, self-perceived safety for provider and patient, inter-disciplinary cooperation, communication and time efficiency during the exercise. Responses using the Likert scale, where 1 was the worst possible and 7 the best possible value, are presented as median with interquartile range (IQR). The number of non-responders is stated where applicable.

Written consent was gathered. The Regional Committee for Medical and Health Research Ethics concluded that the project did not fall under the committee’s mandate (2012/990 A). The Norwegian Social Science Data Service found that the study complied with the requirements of the Personal Data Act (project number 31025). Data were coded and analysed in Excel (Microsoft Corporation, USA) and STATA/SE 11.1 (Statacorp, USA). STROBE (STrengthening the Reporting of OBservational studies in Epidemiology) guidelines for observational research and the SQUIRE (Standards for Quality Improvement Reporting Excellence) guidelines were followed when writing this manuscript [7,8].

## Results

### Cross-sectional survey

We received replies to 219 of 298 (73%) electronic questionnaires sent to all Fire Departments in Norway. The median number of fire fighters in each service was 26 IQR (19, 41). Of these services, 10% consisted of on-call personnel, 74% of deployed personnel and 16% of both. One respondent did not reply to this question. Further results are depicted in tables (c.f. Tables 1 and 2).

**Table 1:** Whom Fire Departments learned the Rapid Extrication chain method from and interdisciplinary training

**Who they learned the Rapid Extrication chain method from?**	
Interdisciplinary cooperation courses	81%
Norwegian Fire Academy	26%
From other fire departments	21%
Other sources	11%
EMS	0,5%
**Do they train the method and with which disciplines?**	
Yes	74%
Training with EMS	72%
Training with Police	36%
Training with motor vehicle salvagers	28%
Training with Helicopter EMS	11%
Training with general practitioners on-call	11%

Note: RE (Rapid Extrication); EMS (Emergency Medical Service).

**Table 2:** Data on protocol, training and real use as provided by the 219 fire stations that responded

	**Passenger car**	**Larger vehicle**
Written protocol		
Yes	151	42
No	61	152
Do not know/no answer	7	25
Number of trainings per year		
Median and IQR	2 (2,3)	1 (1,1)
Do not know/no answer	6	93
Number of interdisciplinary trainings per year		
Median and IQR	1 (1,2)	1 (1,1)
Do not know/no answer	57	161
Number of times used in real situations		
0	35	138
1-10	114	57
11-20	31	2
21-30	14	
31-40	2	
41-50	3	
51-60	3	
Do not know/no answer	17	22

### Feasibility study

In the National Championship all nine teams consisting in total of 63 rescue personnel; 38 fire fighters, 18 EMS personnel (paramedic, nurse or Emergency Medical Technicians (EMT)), six police officers and one motor vehicle salvager consented to participate in the study.

The median time from start until the patient was extricated from car wreckage was 12.5 minutes (11.5,14.2). All teams extricated and transported the patient to the ambulance within 20 min. Further results are depicted in Table 3.

**Table 3:** Participants experience with use of the Rapid Extrication chain method during the National Championship

**Annual training frequency**	**Self – reported security of crew**	**Patient safety**	**Team communication**	**Teamwork**	**Technique function**
2,5 (2,4)	7 (6,7)*	7 (6,7)*	7 (6,7)*	7 (6,7)*	7 (6,7)*

The numbers provided are median. Interquartile range is provided in brackets. *indicates data that are reported using the Likert scale (1 is worst, 7 best).

## Discussion

Our study shows that 95% of the Norwegian fire services that responded have implemented the rapid extrication method on passenger cars and 77% use the technique on larger vehicles. Further, 69% have written protocols for use on passenger cars and 19% for larger vehicles. Participants in the National Championship were satisfied with how the technique functioned and with the security, communication and teamwork. When using RE all teams had the patient extricated and transported to the ambulance within 20 min. Interdisciplinary training was widely in use. In our opinion these are critical factors to prevent sustained hypoxia, uncontrolled bleeding, hypothermia and for the overall survival of the seriously injured trauma patient.

This method can be implemented in all Fire Departments that have the resources to practice and proper equipment (winch on fire truck, chains and hydraulic tools) to perform the method safely. The method might be made more accessible to low and middle income countries by using for example a tree and a tractor instead of two fire engines, as the function of one of the fire engines is to hold the vehicle in place whilst the other pulls. One could also imagine using cheaper tools than hydraulics for cutting of pillars, but before this can be a suggested technique one should look into the potential dangers of using less advanced tools. If this method is successfully implemented using simpler methods it could be a life and morbidity saving method for the millions who annually suffer from road traffic injuries in low-income countries [2]. An animation has been developed showing use of the RE method (Additional file 3).

### Limitations

According to the Directorate for Civil Protection and Emergency Planning there were 298 fire stations in Norway at the time of the study. Of these, 43 are inter-county services and and some of these responders may have replied on behalf of several stations. This would mean a falsely high missing data.

There was a technical error in the electronic questionnaire regarding the question how often they undergo interdisciplinary training of the method on larger vehicles. There was no possibility to reply NIL and we believe this to be the reason for 161 not replying to this question.

The question regarding how many times the method has been used since implementation may include some recall bias as it could be difficult to remember the exact number. The answers of the participants at the National Championship may be influenced by response bias, resulting in falsely many positive answers. Furthermore, responders have a tendency to over-rate their own skills [9].

## Conclusion

The Rapid Extrication chain method is widely in use in Norwegian Fire Departments. Interdisciplinary and regular training of the method can lead to extrication of a critically injured patient in less than 20 minutes. It could prove to be a globally feasible method that is life saving for the critically injured patient.

## Competing interests

The Norwegian Air Ambulance Foundation (NAAF) employs all authors, provides TAS courses free of charge and finances the National Championships in RE. We have no financial interest, only an intellectual interest in disseminating information about TAS courses and the RE method.

## Authors’ contributions

SF, ASJ, JEA, TV and MR participated in the design of the study. TO and JEA performed data collection from the fire stations in Norway. JEA arranged the National Championship. SF and ASJ performed data collection at the National Championship. SF and ASJ performed data analysis. All authors participated in the writing of the manuscript and approved the final version.

## Pre-publication history

The pre-publication history for this paper can be accessed here:

http://www.biomedcentral.com/1471-227X/14/14/prepub

## Supplementary Material

Additional file 1 Rapid extrication animation. Click here for file

Additional file 2 Questionnaire Fire Departments – cross-sectional study. Click here for file

Additional file 3 Questionnaire National Championship participants – feasibility study. Click here for file
