# Supplementary material for: Rapid extrication of entrapped victims in motor vehicle wreckage using a Norwegian chain method – cross-sectional and feasibility study
Source: BMC Emerg Med. 2014 Jul 3;14:14. doi: 10.1186/1471-227X-14-14 (PMC4110241; doi:10.1186/1471-227X-14-14)
Supplement: Additional file 2 — Questionnaire Fire Departments – cross-sectional study. [file 1471-227X-14-14-S2.docx]

**Questionnaire sent to all Fire Departments in Norway:**

Rapid extrication of entrapped victims in motor vehicle wreckage using a Norwegian chain method (translated version)

1) How many fire rescue men/women work in your service?

2) Does your service consist of: 🞐 Deployed or 🞐 On-call personnel?

3) Does your service utilize the rapid extrication technique with chains on:

Passenger cars? 🞐 Yes 🞐 No If no: Why not?

Trucks? 🞐 Yes 🞐 No If no: Why not?

**For those who reply yes to the question above:**

4) When did your service start to utilize the method? Year:

5) Who taught you the method?

- The Norwegian fire academy 🞐

- Other fire services 🞐

- Emergency Medical Service (EMS) 🞐

- Interdisciplinary Emergency Service Cooperation Course (TAS) 🞐

- Norwegian rescue personnel association 🞐

- Other 🞐 Please state whom:

6) How many times per year does your service train rapid extrication on:

Passenger cars:

0 🞐, 1 🞐, 2 🞐, 3 🞐, 4 🞐, 5 🞐, 6 🞐, 7 🞐, 8 🞐, 9 🞐, 10 🞐, 11 🞐, 12🞐, more often 🞐

Larger vehicles:

0 🞐, 1 🞐, 2 🞐, 3 🞐, 4 🞐, 5 🞐, 6 🞐, 7 🞐, 8 🞐, 9 🞐, 10 🞐, 11 🞐, 12🞐, more often 🞐

7) Do you train with other services?

Yes 🞐 No 🞐 Unknown 🞐

If yes:

🞐 Police 🞐 EMS 🞐 General practitioner on-call

🞐 Air ambulance 🞐 Motor vehicle salvage

If yes: How many times per year

Passenger cars:

0 🞐, 1 🞐, 2 🞐, 3 🞐, 4 🞐, 5 🞐, 6 🞐, 7 🞐, 8 🞐, 9 🞐, 10 🞐, 11 🞐, 12🞐, more often 🞐

Larger vehicles:

0 🞐, 1 🞐, 2 🞐, 3 🞐, 4 🞐, 5 🞐, 6 🞐, 7 🞐, 8 🞐, 9 🞐, 10 🞐, 11 🞐, 12🞐, more often 🞐

8) Does your service have written protocol on rapid extrication with chains?

Passenger cars:

Yes 🞐 No 🞐 Unknown 🞐

Larger vehicles:

Yes 🞐 No 🞐 Unknown 🞐

If yes: Could you please send a copy to: hurtigfrigjoring@norskluftambulanse.no

9) Does your service have fire trucks with winch?

Yes 🞐 No 🞐 Unknown 🞐

10) Has your service participated in Interdisciplinary Emergency Service Cooperation Courses (TAS)?

Yes 🞐 No 🞐 Unknown 🞐

If yes, how many times and year of participation?

TAS 1 🞐 Number of times: ______ Year: ______

TAS 2 🞐 Number of times: _______ Year: _______

TAS 2 (repetition) 🞐 Number of times: ________ Year: _______

TAS 3 🞐 Number of times: _______ Year: ______

11) How many times has your service utilized the Rapid Extrication chain method in real situations?

On passenger cars:

0 🞐, 1- 10 🞐, 11-20 🞐, 21-30 🞐, 31-40 🞐, 41-50 🞐, 51-60 🞐, 61-70 🞐, 71-80 🞐, 81-90 🞐, 91-100 🞐, more than 100 times 🞐 unknown 🞐

On larger vehicles:

0 🞐, 1- 10 🞐, 11-20 🞐, 21-30 🞐, 31-40 🞐, 41-50 🞐, 51-60 🞐, 61-70 🞐, 71-80 🞐, 81-90 🞐, 91-100 🞐, more than 100 times 🞐 unknown 🞐
