# Supplementary material for: Rapid extrication of entrapped victims in motor vehicle wreckage using a Norwegian chain method – cross-sectional and feasibility study
Source: BMC Emerg Med. 2014 Jul 3;14:14. doi: 10.1186/1471-227X-14-14 (PMC4110241; doi:10.1186/1471-227X-14-14)
Supplement: Additional file 3 — Questionnaire National Championship participants – feasibility study. [file 1471-227X-14-14-S3.docx]

**Questionnaire presented to participants at the National Championship:**

Rapid extrication of entrapped victims in motor vehicle wreckage using a Norwegian chain method (translated version)

1) How did the method for rapid extrication work?

1🞐 2🞐 3🞐 4 🞐 5🞐 6🞐 7🞐

(1=*Did not work*, 7=*Worked excellent*)

2) How was your safety?

1🞐 2🞐 3🞐 4 🞐 5🞐 6🞐 7🞐

(1=*Very poor*, 7=*Very good*)

3) How was patient safety?

1🞐 2🞐 3🞐 4 🞐 5🞐 6🞐 7🞐

(1=*Very poor*, 7=*Very good*)

4) How did the inter-disciplinary division of responsibilities work?

1🞐 2🞐 3🞐 4 🞐 5🞐 6🞐 7🞐

(1=*Did not work*, 7=*Worked excellent*)

5) How did the inter-disciplinary communication work?

1🞐 2🞐 3🞐 4 🞐 5🞐 6🞐 7🞐

(1=*Did not work*, 7=*Worked excellent*)

6) How time-efficient was the procedure?

1🞐 2🞐 3🞐 4 🞐 5🞐 6🞐 7🞐

(1=*Very inefficient*, 7=*Very efficient*)

7) How many times did you train the last month prior to the National Championship?

Number of times:

**General**

8) What is your profession?

Fire rescue: 🞐 Full-time employed 🞐 Part-time employed

Health: 🞐 Doctor 🞐 Nurse 🞐 Paramedic

🞐 Emergency Medical Technician

Police 🞐

Motor vehicle salvager 🞐

9) How often do you train on the technique?

Number of training sessions last year:

Number of training sessions last month:

10) Does other services participate on the training?

If yes: State which:

11) Has your service participated in Interdisciplinary Emergency Service Cooperation Courses 2 (TAS)? 🞐 Yes 🞐 No

If yes: When (year)?

Has your service participated in Interdisciplinary Emergency Service Cooperation Courses 3 (TAS)? 🞐 Yes 🞐 No

If yes: When (year)?

12) If No on question 11, where have you learned the technique?
